# Heat Shock Proteins: Important Helpers for the Development, Maintenance and Regeneration of Skeletal Muscles

**DOI:** 10.3390/muscles2020014

**Published:** 2023-04-18

**Authors:** Silvia Pomella, Matteo Cassandri, Francesco Antoniani, Samuele Crotti, Laura Mediani, Beatrice Silvestri, Margherita Medici, Rossella Rota, Alessandro Rosa, Serena Carra

**Affiliations:** 1 Department of Oncohematology, Bambino Gesù Children’s Hospital, IRCCS, 00165 Rome, Italy; 2 Department of Clinical Sciences and Translational Medicine, University of Rome Tor Vergata, 00133 Rome, Italy; 3 Department of Radiological Sciences, Oncology and Anatomical Pathology, Sapienza University of Rome, 00161 Rome, Italy; 4 Department of Biomedical, Metabolic and Neural Sciences, University of Modena and Reggio Emilia, 41125 Modena, Italy; 5 Department of Biology and Biotechnologies “Charles Darwin”, Sapienza University of Rome, 00161 Rome, Italy; 6 Center for Life Nano- & Neuro-Science, Fondazione Istituto Italiano di Tecnologia (IIT), 00161 Rome, Italy

**Keywords:** heat shock proteins, muscle differentiation, neuromuscular disease, skeletal muscle plasticity

## Abstract

The skeletal muscle is a highly plastic tissue that shows a remarkable adaptive capacity in response to acute and resistance exercise, and modifies its composition to adapt to use and disuse, a process referred to as muscle plasticity. Heat shock proteins (HSPs), a class of evolutionarily conserved molecular chaperones, have been implicated in the regulation of skeletal muscle plasticity. Here, we summarize key findings supporting the notion that HSPs are important components required to maintain skeletal muscle integrity and functionality. HSPs participate in the transcriptional program required for myogenesis and are activated following muscle exercise and injury. Their dysfunction, either as a consequence of improper expression or genetic mutations, contributes to muscle atrophy and leads to the development of myopathies and peripheral motor neuropathies. Denervation/reinnervation and repeated rounds of nerve degeneration/regeneration have been observed in motor neuropathies, suggesting that an imbalance in HSP expression and function may impair the repair of the neuromuscular junctions. Boosting HSP activity may help preventing muscle atrophy by promoting muscle differentiation and helping the repair of NMJs. Boosting HSP function may also help to combat the development of rhabdomyosarcoma (RMS), a highly aggressive type of pediatric soft tissue sarcoma whose cells have skeletal muscle features but are unable to fully differentiate into skeletal muscle cells.

## 1. Introduction

The skeletal muscle is a heterogeneous and dynamic tissue that originates through a process called myogenesis. The skeletal muscle fibers are innervated by peripheral motor neurons that together form the so-called motor units. The genesis of skeletal muscles occurs during embryonic development, following a sophisticated program that differentiates myogenic precursor cells into functional muscle fibers. The embryonic development of the neuromuscular junctions, the nerve-evoked muscle activity, as well as the trophic factors released by the innervating motor neurons all contribute to muscle development and the formation of functional motor units. Yet, adult organisms maintain the ability to generate new muscle fibers during adulthood and, to some extent, repair dysfunctional motor units [1]. Adult myogenesis relies on the activation of satellite cells, which are adult myogenic progenitors that have the potential to differentiate into new muscle fibers, following a program that is similar to the one that is activated during embryogenesis [2,3]. Thus, the skeletal muscle and the motor units continuously evolve in response to physical activity and inactivity, a process referred to as skeletal muscle plasticity, and more generally motor unit plasticity; these ensure muscle fiber maintenance, repair and remodeling, and as such are fundamental to sustain the muscles’ optimal functionality during adulthood. Loss of skeletal muscle plasticity contributes to muscle atrophy, known as sarcopenia, which corresponds to muscle loss with aging as well as muscle-related diseases [4]. Understanding the molecular players that can promote skeletal muscle differentiation and plasticity and regenerate functional motor units holds promise for the treatment of human diseases characterized by progressive muscular atrophy and aberrant skeletal muscle differentiation, which range from muscular dystrophies, to myopathies, neuromuscular diseases and rhabdomyosarcoma (RMS), a highly aggressive type of cancer that develops from mesenchymal cells that fail to fully differentiate into striated muscle cells [5].

In this review, we will not address the canonical pathways that regulate the process of skeletal muscle differentiation and motor unit development during embryogenesis [6], nor the role of satellite cells in muscle fiber maintenance, repair and remodeling [7,8]. Here, we will rather focus our attention on stress proteins, and in particular, heat shock proteins (HSPs). We will summarize current findings that support the notion that HSPs may participate in skeletal muscle plasticity and the maintenance of functional neuromuscular junctions. Manipulating the expression and activity of HSPs, whose genetic mutations are causes of neuromuscular disorders, may represent a future therapeutic avenue to combat muscular atrophy, regardless of its nature; this muscle atrophy could be prompted by the physiological process of aging, genetic mutations or tissue-specific cancers such as RMS.

## 2. Heat Shock Proteins

Heat shock proteins (HSPs) are evolutionarily conserved proteins that belong to the family of molecular chaperones, which regulate protein folding, maturation and degradation. Maintaining protein homeostasis, the equilibrium between protein synthesis, folding and degradation, is fundamental for life at the cellular and organismal level [9,10]. Thus, given their ability to assist the folding/refolding or clearance of a large variety of proteins (called substrates or clients), HSPs participate in a plethora of biological functions, ranging from cell development, differentiation, apoptosis to response and adaptation to acute and chronic stress [11,12]. Alongside exerting fundamental physiological functions, HSPs have also been involved in disparate disease conditions such as protein conformation disorders, which are characterized by protein aggregation [13], and cancer, where upregulation of HSPs stabilizes overexpressed and mutated cancer proteins, ultimately increasing cancer growth, survival and formation of secondary cancers [14].

Based on their molecular weight, HSPs are classified into the following families: HSP100, HSP90, HSP70, HSP60 and HSP40, with a molecular weight corresponding to 100 kDa, 90 kDa, 70 kDa, 60 kDa, 40 kDa, respectively, and small HSPs, whose molecular weight ranges from 15–35 kDa [10]. Small HSPs, referred to as HSPBs (HSPB1-HSPB10) in mammals, differ from the other HSPs because their activity is independent on ATP, and because they form very dynamic oligomeric ensembles by interacting with themselves and with each other [15]. Based on their expression, HSPs can be classified in two groups: constitutive or inducible and ubiquitous or non-ubiquitous. While some HSPs are constitutively and ubiquitously expressed, such as Hsc70/HSPA8, other members are expressed only following specific stimuli and in a restricted number of cell types, such as testis-specific HSPB9 and HSPB10 [16,17], and muscle-specific HSPB3, which is induced upon myoblast differentiation [18,19]. Amongst the stimuli that can induce HSPs, there are extrinsic and intrinsic factors. Temperature increase is the first stimulus known to induce the expression of HSPs, whose name derives from their serendipitous discovery by the Italian scientist Ferruccio Ritossa back in 1962 [20]. Changes in pH, salinity, osmolarity and oxidative stress, which can promote protein denaturation and unfolding/misfolding, can all induce the expression of several HSPs, a response that is referred to as the heat shock response [12,21,22,23]. Interestingly, these stressors resemble conditions that are experienced by skeletal muscle cells in response to physical exercise, namely, ischemia, oxidation, hyperthermia, glucose deprivation and metabolic acidosis [24]. Therefore, HSPs should not be merely regarded as stress proteins, but rather as essential components required to maintain protein homeostasis from the cellular to the tissue and organismal level.

## 3. Heat Shock Proteins and Skeletal Muscle Differentiation

Our current knowledge about the implication of HSPs in skeletal muscle myogenesis mainly derives from experiments performed in murine C2C12 myoblasts that have been differentiated into myotubes, in which the relative expression level of several HSPs has been evaluated prior to and during differentiation, or in which the expression and/or activity of a given HSP has been inhibited, genetically or pharmacologically, followed by the examination of the differentiation capacity of these modified cells. The importance of HSPs in muscle development and maintenance has been further supported by experimental evidence using animal models such as C. elegans, zebrafish and mice. Finally, the importance of HSPs for the viability and functionality of skeletal muscle cells is further highlighted by the finding that genetic mutations in several HSPs, such as HSPB1, HSPB3, HSPB5, HSPB8, HSP40s (DNAJB4 and DNAJB6) and the HSP70 co-chaperone BAG3, are associated with myopathies and neuromuscular diseases in humans [25,26,27,28,29,30,31,32].

During the early steps of myogenesis, myogenic cells exit the cell cycle to become myoblasts and then fuse into multinucleate myotubes. The key transcription factors that induce muscle cell differentiation are the myogenic regulatory factors (MRFs), namely MyoD, Myf-5, myogenin and MRF-4, which are all myogenic bHLH (basic/Helix–Loop–Helix). The MEF2 (Myocyte Enhancer Factor 2) family of transcription factors cooperates with the MRFs to synergistically activate the expression of key muscle-specific genes [33]. Genes that are targeted by these transcription factors include HSP-coding genes. An MRF-binding site is located in the CRYAB gene, encoding alphaB-crystallin/HSPB5, whose mRNA levels rise during myogenesis [34]. During myogenesis, MyoD upregulates the expression of other small HSPs: HSPB1/Hsp27, HSPB2 and HSPB3 [19]. However, in contrast to HSPB1 and HSPB5, which are expressed also in cycling muscle cells, HSPB2 and HSPB3 are absent in cycling muscle cells, and their expression is specifically induced during muscle cell differentiation, suggesting that these two HSPBs may participate in the muscle differentiation process [18,19]. Beyond the small HSP family, MyoD regulates the expression of other chaperones (Bar-Lavan 2016 PLoS Genet). In particular, upon murine myoblasts differentiation, the expression of Hsp90 alpha and the co-chaperone p23 is inhibited, while the expression of Hsp90 beta and the co-chaperone Aarsd1L is induced by MyoD [35]. The switch in the expression between Hsp90 alpha-p23 and Hsp90 beta- Aarsd1L is required to promote myotube formation in murine cells [35]; meanwhile, pharmacologic inhibition of HSP90 impaired myogenic differentiation and the survival of C2C12 cells, at least in part, via decreasing the levels of MyoD and myogenin [36]. Together, these findings clearly establish a functional implication of HSP90 in muscle development. In agreement, in C. elegans, reducing the expression of hsp-90 and the small heat-shock protein hsp-12.2, which are upregulated by the myogenic transcription factor MYOD, impaired myogenesis and muscle development [37]. Instead, downregulation of Hsp27/HSPB1 expression in developing zebrafish using antisense hsp27 phosphorodiamidate morpholino oligonucleotides did not affect the development of cardiac, smooth, and skeletal muscles [38]. Curiously, HSPB1 depletion specifically impaired the development and growth of craniofacial muscles in zebrafish, suggesting a very specific function for this chaperone that is restricted only to a subset of muscle cells [39]. Concerning the muscle-specific chaperone HSPB3, its depletion in human myoblasts severely affected the muscle transcriptional program, decreasing the expression of key muscle-specific genes such as, e.g., MYOG, ACTA1, DES, coding for myogenin, skeletal muscle-specific actin alpha 1 and desmin, respectively [18]. Moreover, overexpression of HSPB3 in human myoblasts grown under cycling conditions enhanced the expression of genes required for extracellular matrix remodeling, which supports myoblasts’ differentiation [40] as well as satellite cell activation and muscle regeneration [41,42], and is sufficient to induce the differentiation of rhabdomyosarcoma cells [18]. In fact, forced expression of an exogenous HSPB3 in an RMS cell line resulted in fiber-like multinucleated fused structures characterized by increased expression of MYOG and de novo expression of the late marker of skeletal muscle differentiation, *MyH2* [18]. Conversely, overexpression of HSPB2, which is co-upregulated with HSPB3 during myoblast differentiation, did not promote the myogenic transcriptional program [18].

**Figure 1:**
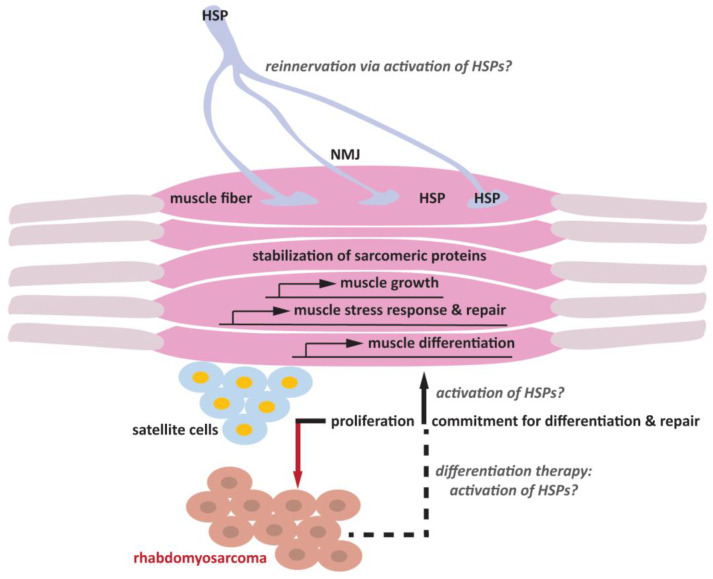
Schematic representation of the putative roles of HSPs in skeletal muscle differentiation and the maintenance of the neuromuscular junction (NMJ).

Recently, a computational analysis of large-scale tissue transcriptomes allowed a comprehensive study of chaperone expression across tissues, highlighting the existence of a muscle-specific signature. In particular, the comparison of the transcriptomic profile of human and C. elegans skeletal muscles identified 58 C. elegans chaperones and 48 homologous chaperones, including HSPs, that were specifically up-regulated in muscles [43]. Together, these experimental and computational data support the notion that HSPs play evolutionarily conserved functions for the development and maintenance of skeletal muscles [43]. However, there is only partial redundancy between the various HSPs, and their function in the context of muscle differentiation cannot be generalized. Importantly, we still have a limited understanding of how the upregulation of specific HSPs participates in myogenesis, and what their specific molecular targets are (Figure 1).

## 4. Heat Shock Proteins, Muscle Activity and Resistance Training

The skeletal muscle, which is made of fast and slow skeletal muscle motor units, is a highly plastic tissue that shows a remarkable adaptive capacity in response to acute and resistance exercise, and modifies its chemical and structural composition to adapt to use and disuse. Skeletal muscles undergo hypertrophy and increase their mass when subjected to strength training, increase their mitochondrial number when subjected to endurance training and, conversely, undergo atrophy in the absence of exercise or as a result of a disease state that directly impairs muscle maintenance. HSPs have been implicated in the regulation of skeletal muscle plasticity.

Exercise represents a physiological stress that is accompanied by changes in the temperature, pH, ion concentration and oxidation, as well as glucose deprivation and a decrease in intramuscular glycogen and calcium concentration, all factors that can trigger the expression of HSPs [21]. Of note, heat shock activates the muscle transcriptional program by enhancing the expression of key myogenic transcription factors such as myocyte enhancer factor 2D (MEF2D), myogenic differentiation factor 1 (MYOD1), myogenic factor 5 (MYF5) and myogenic factor 6 (MYF6), increasing myotube formation [44] and thus providing a direct link between HSPs and muscle build-up and maintenance.

Initial studies in mice and humans focused on HSP70, demonstrating that skeletal muscles upregulate its expression following exercise [45,46]. In humans, there are 13 genes encoding for distinct HSP70 proteins that according to the new nomenclature are named HSPA1A, HSPA1B, HSPA1L, HSPA2, HSPA4, HSPA4L, HSPA5, HSPA6, HSPA7, HSPA8, HSPA9, HSPA12A and HSPA14 [47]. HSPA1A, also known as HSP72/HSP70-1, and HSPA1B, also known as HSP70-2, are strongly upregulated in the skeletal muscle and are generally referred to in the older literature as HSP70 or HSP72. A large body of evidence demonstrates that HSP70 upregulation in the skeletal muscle occurs not only after exercise but also following muscle injury and upon muscle regeneration [48,49,50,51]. By contrast, HSP70 expression is reduced following periods of muscle inactivity that decrease muscle mass. The functional significance of the changes in HSP70 expression for skeletal muscle plasticity comes from studies using transgenic mice. On the one hand, HSP70 overexpression in mice increased muscle mass and muscle regeneration after injury; on the other hand, HSP70 knockout in mice decreased muscle regeneration and recovery after injury. Thus, HSP70 is not only required to maintain skeletal mass and integrity, but also to promote its regeneration and recovery, protecting it against wasting and damage [49,51,52].

Besides HSP70s, a number of studies focused on the other master cellular chaperone, HSP90. Both acute and chronic exercises induce the expression of HSP90, along with HSP70. The extent of HSP90 induction depends on the type, strength and duration of the physical exercise. Similar to HSP70 upregulation, HSP90 upregulation is thought to participate in the restoration of protein homeostasis, as well as in the activation of muscle repair and regeneration during the recovery process [48]. Using cellular and C. elegans models, it was demonstrated that HSP90 participates in the maintenance of muscle structures, at least in part, through interaction with and stabilization of myofilaments [53].

Following studies have shown that along with HSP70 mRNAs, expression of other chaperones such as HSPB1 and HSPB5 was also upregulated in humans subjected to repeated contractions [54,55]. Following eccentric exercise, besides changes in the expression levels of HSPs, changes in their subcellular localization have also been observed, with relocalization of, e.g., HSP70, HSPB1 and HSPB5 from the cytoplasm to the cytoskeleton, wherein these proteins are thought to prevent protein denaturation and stabilize sarcomeric proteins [56]. In agreement with a potential implication of HSPs in muscle plasticity, overexpression of HSP72 and Hsp27/HSPB1 was sufficient to preserve muscle strength and mass in disuse atrophy [50,57].

Finally, a recent proteomic study conducted using muscle biopsies from participants chosen based on their physical exercise identified several chaperones that are upregulated upon daily activity. The 20 upregulated chaperones include members of the HSP90, HSP70, HSP40 and HSPB families, such as for example HSP90AA1, HSP90AB1, HSP90B1, HSP72, HSPA2, HSPA8, HSPA9, DNAJB4 and HSPB1 [58].

Together, these data demonstrate that HSPs can act as pro-myogenic factors and support the hypothesis that the activation of the Heat Shock Response is critical for the remodeling/adaptation of skeletal muscles to high-force exercise, as well as to muscle stress and injury, preserving muscle structure and function from age-related damage [54,55,58].

## 5. Heat Shock Proteins and Muscle Disease

The most compelling evidence supporting the direct function of HSPs in the maintenance of skeletal muscles comes from genetic studies that identified muscular diseases caused by mutations in HSP genes.

For example, mutations in the genes coding for DNAJB4 are associated with adult-onset myofibrillar myopathy with early respiratory failure [25], while mutations in the gene coding for DNAJB6 have been associated with limb-girdle muscular dystrophy (LGMD) [31,59], myofibrillar myopathy [60] and juvenile-onset proximal-distal myopathy [61]. The P209L mutation in the gene coding for the HSP70 co-chaperone BAG3 was associated with severe dominant childhood muscular dystrophy associated with cardiomyopathy, and respiratory failure [28]. Later, more than 20 different frameshift mutations with premature stop codons, missense and nonsense mutations in the BAG3 gene have been identified and associated with myofibrillar myopathy or dilated cardiomyopathy [62]. Of note, BAG3 is a nucleotide exchange factor that regulates substrate binding and release by HSP70 through the modulation of its ATP-consuming cycle [63]. Neuropathy was documented in cases of BAG3-associated myofibrillar myopathy, followed by the identification of BAG3 mutations in patients affected by sensorimotor neuropathy [64]. In analogy, mutations in the HSPB8 gene were initially identified as causative for peripheral motor neuropathy [26] and have recently been associated also with distal myopathy [32] and with rimmed vacuolar myopathy [30,65]. Similar to BAG3 and HSPB8, mutations in the genes coding for DNAJB2, HSPB1 and HSPB3 have been associated with distal hereditary motor neuropathy (dHMN), including Charcot–Marie–Tooth disease [30]. Next, mutations in the HSPB5 gene have been linked to dominantly inherited myofibrillar myopathy, hypertrophic cardiomyopathy and only sporadically with neuropathy [30,66]. Please see [30] and [67] for a detailed description of chaperone mutations linked to neuromuscular diseases. Finally, a series of rare variants in the HSPB1 and HSPB3 genes have been identified, namely HSPB1-S135A, HSPB3-G67S, HSPB3-R116X [68] and HSPB1-R127W, HSPB1-D149A and HSPB1-T151P [69]; these rare variants are likely pathogenic, and it has been suggested that they may represent a burden in amyotrophic lateral sclerosis (ALS).

The implication of HSPs in the maintenance of the skeletal muscles and, more generally, of the neuromuscular system, has been further corroborated by functional studies showing that upregulation of specific chaperones can protect against muscle atrophy in muscle diseases due to genetic mutations in other non-HSP coding genes. For example, overexpression of HSP72 in dystrophic mice improved body strength and muscular endurance, similar to pharmacological upregulation of HSP72 with BGP-15; these data support the notion that HSP72 induction may exert protective functions and slow-down muscle wasting and atrophy in Duchenne muscular dystrophy (DMD) and other related muscle disorders [70].

The protective effects exerted by HSPs at the level of muscles is further supported by overexpression studies. Briefly, overexpression of HSP70 in mice attenuated skeletal muscle damage induced by cryolesioning [71] and by exercise [72], and attenuated sarcopenia and improved the structural and functional recovery of skeletal muscles from atrophy [73]. In addition, overexpression of Hsp27 (via electrotransfer into the soleus muscle of rats) attenuated skeletal muscle disuse atrophy [57], and overexpression of mitochondrial HSP10 in transgenic mice prevented contraction-induced damage and preserved muscle force generation [74]. In summary, together these data support the notion that HSP upregulation may be beneficial to combat physiological muscle aging and delay the progression of muscle atrophy in disease states.

## 6. Heat Shock Protein Muscle-Specific Targets

The majority of the evolutionarily conserved muscle chaperones, whose expression is specifically upregulated in this tissue compared to other tissues, participate in the folding of well-known muscle structural proteins, hinting at stabilization of sarcomeres as a canonical mechanism through which HSPs (and co-chaperones) participate in muscle maintenance.

The sarcomere of skeletal muscles is composed of three filamentous proteins: thick filaments of myosin, thin filaments of actin, and titin filaments. Titin acts as a molecular spring, ensuring viscoelastic properties to the myofilaments; it also participates in sarcomere assembly, mechano-sensing and signaling, and contributes to passive forces [75]. Instead, actin and myosin are responsible for the force development of the sarcomere, producing muscular movements [76]. Sarcomere proteins are constantly subjected to mechanical forces that can favor their misfolding and aggregation. In particular, the giant protein titin, which spans from the Z disc to the M line, with more than 3000 kDa [77], unfolds upon sarcomere stretching, thereby exposing hydrophobic residues that can lead to titin misfolding and aggregation [78]. Several HSPs have been proposed to bind to titin and stabilize it, including HSPB5 [79], HSPB1 [38,80], and HSP90 [81,82], ultimately protecting myofibrils from stress-induced degradation.

In addition, the chaperone complex composed of Hsc70/HSPA8, its co-chaperone BAG3, and HSPB8 has been shown to localize to Z-discs and play a fundamental role in Z-disc maintenance by preventing protein aggregation [83]. Amongst the sarcomeric proteins targeted by the HSPB8-BAG3-Hsc70 chaperone complex is filamin C, an actin filament crosslinking protein that localizes to Z-discs to maintain their organization [83,84]. Following this discovery, other chaperones such as HSPB1 [85] and HSPB7 [86] were shown to bind to and stabilize filamin C, indirectly participating in its mechanosensing functions. Importantly, their dysfunction or depletion has been associated with Z-disc disorganization in animal models and with the progression of myopathies in humans [28,83,86,87].

Other important structural proteins that are targeted by chaperones are actin, intermediate filament proteins and myosin. A large body of evidence collected using different cell types, including cancer cells, demonstrates that actin filaments, as well as intermediate filaments are stabilized by HSPB1, HSPB5 and HSPB7 [88,89,90,91,92,93]. In skeletal muscle cells, the lack of stabilization of desmin and actin filaments due to HSPB dysfunction (or genetic mutation) has been implicated in protein aggregation, aberrant sarcomere assembly [94,95], as well as disease progression [96]. Concerning myosin, its replacement is promoted by HSP90. Besides surveying myosin folding, HSP90 induces the MYH gene expression, enhancing myosin cytosolic content and ensuring its proper assembly in the muscle sarcomere [97]. Thus, collectively, HSPs exert a critical protein quality control function on sarcomeric proteins, protecting them from unfolding and aggregation due to mechanical stress and exercise and promoting their clearance, which is essential to maintain muscle integrity. Yet, their mechanism of action seems to extend beyond the sole chaperone activity, with some HSPs also being able to indirectly modulate gene expression and signaling pathways implicated in muscle plasticity. For example, the heat-inducible members of the HSPA, HSPC and HSPB families can interact with signaling factors involved in muscle hypertrophy and atrophy [98]. HSP70 inhibits NF-kappaB and FOXO signaling, thereby limiting physiological disuse muscle atrophy while promoting muscle regeneration [50]. Similarly, overexpression of HSPB1 decreased muscle wasting during disuse atrophy through inhibition of NF-kB, MuRF1, and MAFbx [57]. In addition, HSP70 interacts with and stimulates the Akt-mTOR signaling pathway, a critical regulator of skeletal muscle mass [99].

Finally, myogenic transcription factors are targeted by the two main master chaperones in cells: HSP70s and HSP90s. For example, Hsc70 and HSP70 interact with MK2, a substrate of p38 mitogen-activated protein kinase (p38MAPK), ultimately stabilizing it. This, in turn, promotes the expression of E47, a splice product of the E2A gene that interacts with MyoD to activate transcription, as well as myocyte enhancer binding factors MEF2A and MEF2C, and BAF60, a subunit of the chromatin-modifying enzyme SWI/SNF that participates in muscle determination and renewal [100]. Instead, HSP90 stabilizes MyoD and MyoG and promotes the conformational change of MyoD from an inactive to an active state [36,101]. Concerning sHSPs, αB-crystallin/HSPB5 affects cell cycle exit and decreases MyoD levels, promoting its ubiquitination and degradation [102], while HSPB3 indirectly stabilizes myogenin, enhancing myogenesis [18].

## 7. Future Research Direction: Regeneration of Muscle Fibers and Neuromuscular Junction

Cytoskeletal maintenance, protein quality control at the sarcomeres, maintenance of Z-disc organization and their general anti-aggregation functions are undoubtedly implicated in the protective functions exerted by HSPs at the level of skeletal muscles. Exercise induces muscle damage and activates processes aimed at regenerating and repairing the damaged fibers [103]. In addition, exercise stimulates the neuromuscular junctions (NMJs), eliciting functional and morphological remodeling to adapt the response of the neuromuscular system to endurance and resistance training [104,105].

The repair of the damaged muscle relies on the activation of the satellite cells and their differentiation to generate new myofibers, with mechanisms similar to the ones that are in place during embryonic muscle development [106]. As previously discussed, several HSPs are induced during and participate in the differentiation program, with as yet unclear mechanisms. Whether HSPs actively promote adult myogenesis in response to exercise-induced damage or injury, and whether they participate in NMJ plasticity, is still poorly understood. However, the neuromuscular system as a whole, rather than skeletal muscle cells per se, seems to be sensitive to HSP dysfunction. In fact, mutations in genes coding for several HSPs (e.g., HSPB1, HSPB3, HSPB8 and DNAJB6) are causative not only for myopathies, but also for distal hereditary peripheral neuropathies, which are characterized by motor neuron degeneration. Of note, active denervation/reinnervation and repeated rounds of nerve degeneration/regeneration have been observed in distal hereditary motor neuropathies such as Charcot–Marie–Tooth (CMT) type 2 [107], and such findings have been extended to other motor neuron diseases such as ALS. Based on these data, neuromuscular junction (NMJ) pathology has been proposed to represent an early event that precedes and contributes to motor neuron loss both in ALS and CMT [108,109,110,111,112].

Thus, future lines of research should focus on clarifying to what extent HSPs may participate in the reinnervation and regenerative processes that occur in response to exercise and damage, by promoting the differentiation of skeletal muscle cells and the morphological adaptation of NMJs. Although we have known for a long time that most HSPs are widely expressed in the nervous system, wherein they seem to participate in neuronal differentiation, neurotransmission and the maintenance of synapses [113,114,115,116], our knowledge about HSP functions at the level of the NMJ and in the regenerative processes of the motor units is still in its infancy, and has been characterized only for a small subset of chaperones. For example, HSP70 is induced upon muscle injury and during muscle re-growth, and its loss severely impairs muscle regeneration [51]. Conversely, overexpression of HSP70 in mice protects against skeletal muscle atrophy [117], while hyperthermia, which induces a global heat shock response, including HSP70 upregulation, protects skeletal muscles from oxidation and potentiates re-growth after immobilization [118]. These muscle regenerative effects of HSP70 have been in part ascribed to its pro-differentiation function [100]. In addition, injection of recombinant human HSP70 delayed motor neuron degeneration, preserved myelinated peripheral axons and arrested denervation in a mouse model of ALS [100]. Forced expression of HSPB1 ameliorated nerve repair (axonal regeneration) after sciatic nerve injury [119], while HSP90 was shown to participate in NMJ development by regulating acetylcholine receptor (AChR) cluster formation and maintenance [120,121]. DNAJB2, whose genetic mutations are associated with CMT type 2 [29,122], is expressed in regenerating fibers and at the postsynaptic side of NMJs [123]. DNAJB2 was suggested to regulate protein turnover in the skeletal muscle, but we currently ignore its relevance to NMJ function, plasticity and maintenance. Finally, a general RNAi approach in Drosophila melanogaster identified a new set of chaperones, including, e.g., HSP26/CG4183, HSP60/CG12101, Hsc70/CG8542 and HSP90A1/CG1242, which is required for proper NMJ structural organization in Drosophila melanogaster [124]. These data were corroborated by a recent work in which, using NMJ as a model to study synapse formation, the Drosophila melanogaster HSP23 and HSP26 proteins, as members of the small HSP family, were shown to shape the NMJ number and activity during development [125].

Together with earlier data identifying HSPs as direct targets of the key myogenic transcription factors MRFs and MyoD, and considering that mutations in these chaperones are causative for neuromuscular diseases characterized by NMJ degeneration and muscle atrophy, these data highlight the need to better understand whether/how HSPs participate in the maintenance and repair of NMJ (Figure 1). A detailed understanding of the HSP molecular functions in the processes of NMJ and skeletal muscle repair and reinnervation has the potential to pinpoint novel biomarkers and molecular targets for future and innovative therapeutic approaches. This type of research has been hampered by the technical limitations involved in modeling the neuromuscular system in vitro, thereby restricting the experimental design to either single cell cultures (skeletal muscle cells, satellite cells or pure embryonic neuronal populations) or two-dimensional cell culture systems (2D) that do not mimic the complex organization of the neuromuscular system. An improvement over conventional 2D systems has been demonstrated with more complex 3D models of neurodegenerative and neurodevelopmental diseases such as Alzheimer’s disease and microcephaly [126]. These paradigmatic examples suggest that moving from conventional 2D to 3D cultures could significantly improve the production of more physiological in vitro models of human development and disease. Very recently, the Gouti lab developed a human 3D neuromuscular organoid (NMO) containing both the muscle and the nerve compartments of the neuromuscular system [127]. Generation of NMOs is based on the induction of differentiation of pluripotent stem cells into a bipotent axial population of neuromesodermal progenitors (NMPs), which can recapitulate the development of the spinal cord neural and mesodermal lineages at the same time. This results in complex 3D organoids in which different cell types comprising spinal cord neurons, skeletal muscle and terminal Schwann cells interact and self-organize to form functional NMJs. Importantly, NMOs are reproducible and amenable to manipulation and functional testing. NMOs have been successfully used to recapitulate myasthenia gravis, an autoimmune disorder of the NMJ, thus demonstrating the potential of this 3D system for modeling neuromuscular diseases [127]. Using this cutting-edge technology, it will be possible to understand how depletion of specific chaperones and their specific disease-causing mutations affect the NMJ’s development and function, pinpointing the steps at which these chaperones are critical: those of differentiation, maintenance and/or regeneration.

## 8. Skeletal Muscle Differentiation as a Potential Therapeutic Avenue for Rhabdomyosarcoma

Rhabdomyosarcoma (RMS), the most common soft-tissue sarcoma of children and adolescents, has been molecularly divided into two major subgroups: fusion-positive (FP-RMS), harboring the balanced chromosomal translocations, t(2;13)(q35;q14) or t(1;13)(p36;q14), that result in expression of fusion oncoproteins PAX3 or PAX7-FOXO1, and fusion-negative (FN-RMS), which lacks chromosomal translocations but is characterized by a few genomic mutations mainly affecting RAS pathway members (50% of cases), and the *BCOR* (15%), *NF1* (15%), and *TP53* (13%) genes [128,129].

The current standard of care for RMS consists of surgical resection with the addition of chemotherapy and radiotherapy, whilst immunotherapies have not shown an effect (Hawkins, Miwa). Nevertheless, despite aggressive multimodal therapies, the 5-year survival rate for patients with metastatic or recurrent disease remains poor [130].

RMS is unable to complete terminal skeletal muscle differentiation, although it does express the myogenic transcription factors myoblast determination protein 1 (*MYOD)* and myogenin (*MYOG)*; hence, RMS cells are locked in various stages of differentiation of skeletal muscle development [131,132]. Thus, given the developmental nature of RMS tumors, there is a large interest in investigating molecular mechanisms underpinning the differentiation blockade, and substantial efforts have been directed toward identifying and developing strategies to restore the physiological differentiation steps.

To date, few differentiation therapies have been successfully attempted in RMS in vitro and in vivo, each targeting different molecular players that control skeletal muscle differentiation or cell proliferation. Among them, the MEK/ERK inhibitor trametinib has shown striking and promising effects in a preclinical model of FN-RMS, by decreasing RMS cell viability and slowing tumor growth [133]. Particularly, trametinib treatment leads to the release of the ERK2-dependent transcriptional stalling of RNA polymerase II at the *MYOG* genomic locus, allowing the expression of late myogenic genes and thus restoring differentiation [133]. Furthermore, the pharmacological inhibition of the methyltransferase EZH2, the catalytic subunit of the Polycomb repressive complex 2 (PRC2), through either S-adenosylhomocysteine hydrolase inhibitor 3-deazaneplanocin A (DZNep) or MC1945, induces cell cycle arrest and promotes myogenic differentiation in FN-RMS [134]. The hampered enzymatic function of EZH2 reduced the trimethylation of lysine 27 on histone 3 (H3K27me3), a repressive histone mark, which results in an increased expression of its direct repressed target miR-101 [135] and of late differentiation genes *MYOG*, creatine kinase M-type (*CKM*), and the myosin heavy chain (*MHC*) [134]. Recently, Laubscher et al. demonstrated that BRG1-targeting compounds, such as ACBI1, a BRG1 degrader, induce transcriptional activation of the skeletal muscle differentiation program associated with MYCN enhancer invasion at myogenic target genes, which results in myogenic differentiation [136]. Mechanistically, the catalytic subunit BRG1 (encoded by *SMARCA4*) has been found overexpressed in FP-RMS compared to normal skeletal muscle, and it has been demonstrated to be involved in the maintenance of cell proliferation status of FP-RMS [136]. Next, a recent clustered regularly interspaced short palindromic repeats (CRISPR)-based phenotypic screen pointed out the histone deacetylase 3 (HDAC3) as a key mediator of the suppression of the differentiation of RMS [137]. Specifically, the complex NCOR/HDAC blocks MYOD-mediated activation of myogenic differentiation. Given these data, and the discouraging results of pan-HDAC inhibitors in clinical trials of solid tumors, Phelps et al. suggested the development of selective HDAC3 inhibitors as a promising option for differentiation therapy in RMS [137]. In parallel, our group discovered that the transcription factor SNAI2, overexpressed in RMS, binds to genomic key sites of FN-RMS and acts as a repressor of the MYOD-dependent program, thus impairing the terminal differentiation [138]. Since SNAI2 behaves as a downstream player of the MEK-ERK pathway (its knockdown resembles MEK inhibition-dependent myogenic differentiation) it may represent a promising target [138]. Lately, we have also investigated the role of the small heat shock protein HSPB3, induced during muscle differentiation [19], in the context of RMS tumors. HSPB3 exerts pro-differentiation functions in FN-RMS when ectopically expressed by inducing the expression of terminally differentiating genes such as MYOG and MHC at transcriptional and protein levels [18]. Of note, the *HSPB3* levels are markedly lower in FN-RMS cell lines compared to skeletal muscle myoblasts, and *HSPB3* expression increases during muscle differentiation; hence, pharmacological strategies to increase HSPB3 expression could be an innovative and promising approach to develop a new differentiating therapy for RMS (Figure 1).

All this evidence highlights the urgent need for the improvement of therapies able to restore the signaling network involved in skeletal muscle differentiation in RMS, suggesting that overcoming arrested myogenic development could represent one of the most effective therapeutic strategies to improve RMS patient outcomes. Of note, the HSPs that participate in skeletal muscle differentiation and maintenance have been localized to the nucleus, in which they can cooperate to regulate the transcription of specific genes. For example, HSP70 acts as a corepressor with Heat Shock Factor 1 (HSF1), a stress-inducible transcription factor that induces the expression of HSPs and represents the core component of the heat shock response [139]; this, in turn, inhibits the Ras-induced transcription of the c-fos gene [140], and both Ras and c-fos can influence myogenesis [141]. HSP90 instead associates with several steroid aporeceptor transcription factor complexes, and regulates the DNA binding activities of certain transcription factors, including MyoD, ultimately controlling chromatin accessibility [142]. Future studies will uncover whether upregulation of muscle-specific chaperones such as HSPB3 and other HSPs that play a role in the maintenance of skeletal muscle cells may represent a novel strategy to promote terminal skeletal muscle differentiation in the context of RMS.

HSPs are expressed in satellite cells, muscle cells and motor neurons. Under physiological conditions, satellite cells are quiescent. Upon injury, satellite cells proliferate to repopulate the satellite cell niche, and a fraction of cells differentiate into myogenic precursors, which subsequently convert into myoblasts that fuse to generate myotubes and myofibers and repair the damaged muscle fiber. Muscle activity is regulated by the NMJ, a specialized synapse that connects the motor neurons and the skeletal muscle fibers. Upon aging and damage, as well as under pathological conditions, episodes of denervation contribute to skeletal muscle atrophy. Potentiating the expression of HSPs can promote muscle cell differentiation through yet unclear mechanisms that may include induction of the expression of genes involved in muscle build-up and maintenance (grey question marks). HSPs are key players of the cellular stress response that may promote muscle repair, also by stabilizing sarcomeric proteins, thererby protecting them from unfolding and aggregation due to mechanical stress and exercise. In addition, HSPs such as HSPB3 can also promote the differentiation of rhabdomyosarcoma-derived cells, offering a potential therapeutic strategy (grey question marks). Potentiating the expression of HSPs, whose mutations are linked to myopathies and peripheral neuropathies, may promote muscle reinnervation, helping to restore and maintain muscle activity during aging (grey question marks).

## Data Availability

Not applicable.
